# Telemedicine Consult for Shortness of Breath Due to Sympathetic Crashing Acute Pulmonary Edema

**DOI:** 10.21980/J8HS86

**Published:** 2023-01-31

**Authors:** Derek Jacob Carver Hunt, Kevin McLendon, Carl Johns, Daniel Crane

**Affiliations:** * Merit Health Wesley, Department of Emergency Medicine, Hattiesburg, MS

## Abstract

**Audience:**

This simulation is appropriate for senior and junior emergency medicine residents.

**Introduction:**

Shortness of breath is a very common presentation in the emergency department and can range from mild to severe as well as a chronic or acute onset. In sympathetic crashing acute pulmonary edema (SCAPE), patients typically present with acute onset of dyspnea occurring within minutes to hours and have significantly elevated blood pressure.^1^ The condition of SCAPE falls into the spectrum of acute heart failure syndromes such as fluid overload pulmonary edema and congestive heart failure exacerbation.^1^

**Educational Objectives:**

At the completion of the simulation and debriefing, the learner will be able to: 1) recognize the physical exam findings and presentation of SCAPE, 2) utilize imaging and laboratory results to further aid in the diagnosis of SCAPE, 3) initiate treatments necessary for the stabilization of SCAPE, 4) demonstrate the ability to assist with the stabilization and disposition of a patient via tele-medicine as determined by the critical action checklist and assessment tool below, 5) interpret the electrocardiogram (EKG) as atrial fibrillation with rapid ventricular response (AFRVR), and 6) recognize that SCAPE is the underlying cause of AFRVR and continue to treat the former.

**Educational Methods:**

This simulation was performed using a high-fidelity mannequin. In order to simulate the telemedicine aspect, the learner evaluated the patient using a video conferencing interface while the two confederates were present with the high-fidelity mannequin. A debriefing session was held immediately after the simulation.

**Research Methods:**

The educational content was evaluated by debriefing and verbal feedback provided immediately after the case. Additionally, a survey was emailed to participants and observers of the case to provide qualitative feedback.

**Results:**

Post-simulation feedback was overall positive with participants and observers. Participants and observers felt this was a safe and realistic simulation of SCAPE and provided them with the opportunity to practice rapid recognition and treatment of this condition.

**Discussion:**

Sympathetic crashing acute pulmonary edema falls into the spectrum of acute heart failure disorders, and rapid recognition and stabilization is vital for the patient’s survival. This simulation case provided learners of all levels the chance to assess and treat a life-threatening condition with limited information in a safe and effective learning environment. The telemedicine component was used while conducting weekly didactics via zoom during the COVID-19 pandemic. Simulation is a large component of our didactic curriculum and implementing the telemedicine component into this case was worth the effort. It is important to familiarize our residents with telemedicine since we expect that it will become a larger part of the practice of emergency medicine in the future, allowing board-certified emergency medicine physicians to assist in providing care in rural emergency departments and smaller hospitals that may be staffed with less experienced providers.

**Topics:**

Medical simulation, tele-medicine, pulmonary edema, respiratory distress, cardiac emergencies, resuscitation.

## USER GUIDE

**Table t1-jetem-8-1-s1:** 

List of Resources:	
Abstract	1
User Guide	3
Instructor Materials	6
Operator Materials	14
Debriefing and Evaluation Pearls	16
Simulation Assessment	20

[Table t1-jetem-8-1-s1] **Learner Audience:**

Junior and Senior Emergency Medicine Residents

**Time Required for Implementation:**

Instructor Preparation: 20–30 minutes

Time for case: 15 minutes

Time for debriefing: 15–20 minutes

**Recommended Number of Learners per Instructor:** 1–2

**Topics:**

Medical simulation, tele-medicine, pulmonary edema, respiratory distress, cardiac emergencies, resuscitation.

**Objectives:**

At the completion of the simulation and debriefing, the learner will be able to:

1. Recognize the physical exam findings and presentation of SCAPE.
2. Utilize imaging and laboratory results to further aid in the diagnosis of SCAPE.
3. Initiate treatments necessary for the stabilization of SCAPE.
4. Demonstrate the ability to assist with the stabilization and disposition of a patient via telemedicine as determined by the critical action checklist and assessment tool below.
5. Interpret the electrocardiogram (EKG) as atrial fibrillation with rapid ventricular response (AFRVR).
6. Recognize that SCAPE is the underlying cause of AFRVR and continue to treat the former.

### Linked objectives and methods

Shortness of breath and respiratory distress are common emergency department presentations. Occasionally, these symptoms are due to pulmonary edema or SCAPE, which requires rapid recognition and treatment. This case allows learners to review the presentation of SCAPE based on physical exam findings, vital signs, radiographic imaging, and laboratory results (objectives 1 and 3). The learner will then initiate medicinal and respiratory support treatments and demonstrate an understanding of the mechanism of action (objective 2). The tele-medicine format of this simulation case was chosen because our weekly didactic sessions were moved to a video conferencing format due to the COVID-19 pandemic. In our region, tele-medicine is frequently used because there are many critical access hospitals in our predominantly rural state. This case will give the learner the ability to practice telemedicine and demonstrate the ability to assist in patient resuscitation without having direct patient contact (objective 4). It will also assess their ability to interpret an EKG (objective 5), but the learner should recognize that AFRVR is being caused by SCAPE and should be treated with respiratory support and nitrates instead of rate or rhythm control medications (objective 6).

### Recommended pre-reading for instructor

1. Agrawal N, Kumar A, Aggarwal P, Jamshed N. Sympathetic crashing acute pulmonary edema. *Indian J Crit Care Med*. 2016;20(12):719-723. doi:10.4103/0972-5229.195710
2. We recommend instructors to review current literature on the diagnosis and treatment of SCAPE as well as ACEP Clinical Guidelines on Acute Heart Failure Syndromes. Additional sources are listed in the references/suggestions section.

### Results and tips for successful implementation

This simulation was written to be performed during virtual didactics while using the ZOOM video conferencing platform during the COVID-19 pandemic. In order to perform the telemedicine component, a laptop was placed on a table at the foot of the mannequin’s bed. The laptop was logged into the ZOOM didactic session to allow the participant and observers to see the mannequin, cardiac monitor, and the two confederates. One second-year resident participated in the simulation case while the remainder of the residents and faculty observed. This format allowed us to demonstrate a scenario that simulates SCAPE and provides the stimulus needed during an emergent tele-medicine consult. To perform this simulation as written, we would recommend conducting the case over a video conferencing format with one to two learners and two confederates. The instructor and confederates should be with the high-fidelity mannequin and accessible over the video conference. This case could also be performed without the video conferencing format and could also be utilized as an oral board case. Learners were evaluated by the instructor(s) based on their ability to correctly diagnose and treat the patient.

Participant and observer feedback was overall very positive, and they did not recommend any significant changes to the case design or format. This case was initially performed during the COVID-19 pandemic while all didactic sessions were conducted via video-conferencing. Because of this, only one junior resident was able to perform the case in the tele-medicine format during a didactic session. Of the seven responses received, one was from the participant and six were from observers. The survey was sent immediately after didactics concluded via Google Forms through the residency programs GroupMe messaging group, and all responses were anonymous. Results of the survey are below.[Fig f1-jetem-8-1-s1][Fig f2-jetem-8-1-s1][Fig f3-jetem-8-1-s1][Fig f4-jetem-8-1-s1][Fig f5-jetem-8-1-s1][Fig f6-jetem-8-1-s1][Fig f7-jetem-8-1-s1]

**Figure f1-jetem-8-1-s1:**
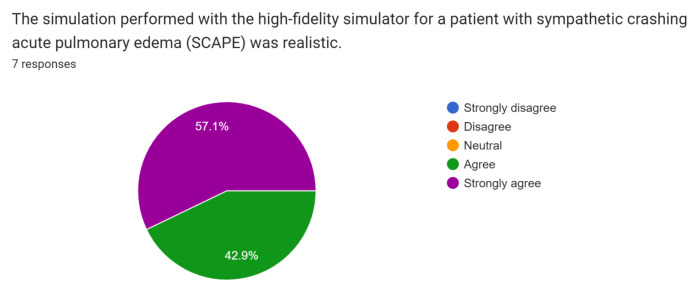


**Figure f2-jetem-8-1-s1:**
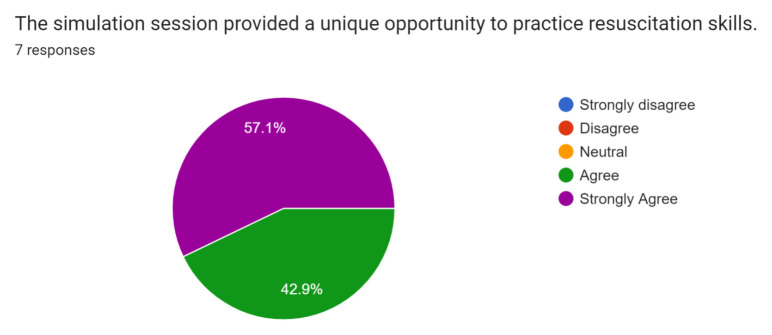


**Figure f3-jetem-8-1-s1:**
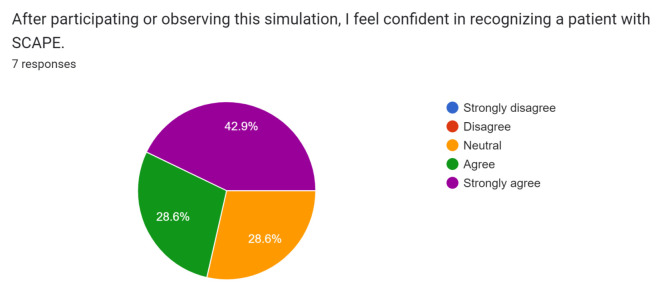


**Figure f4-jetem-8-1-s1:**
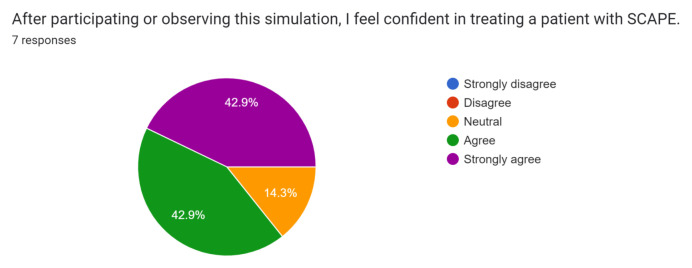


**Figure f5-jetem-8-1-s1:**
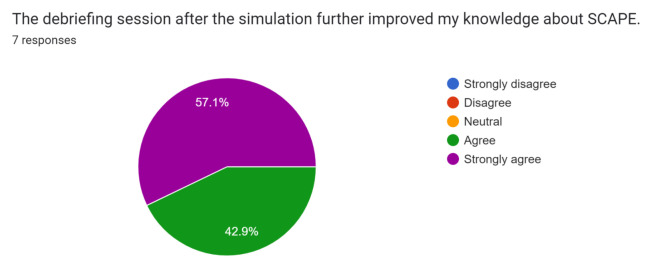


## Supplementary Information



















## Figures and Tables

**Table t2-jetem-8-1-s1:** 

#1	Complete blood count (CBC)
#2	Basic Metabolic Panel (BMP)
#3	Brain Natriuretic Peptide
#4	High Sensitivity Troponin
#5	Coagulation Studies
#6	Arterial Blood Gas (ABG)
#7	Chest Radiograph (CXR)
#8	Electrocardiogram (ECG)

**Table t3-jetem-8-1-s1:** 

**Vitals:**	Heart Rate (HR) 132	Blood Pressure (BP) 245/180	Respiratory Rate (RR) 32
	Temperature (T) 99°F	Oxygen Saturation (O2Sat) 85% on 2L/min	

**Table t4-jetem-8-1-s1:** 

*Complete blood count (CBC)*	
White blood count (WBC)	9.8 x1000/mm^3^
Hemoglobin (Hgb)	14.5 g/dL
Hematocrit (HCT)	33.0%
Platelet (Plt)	385 x1000/mm^3^

**Table t5-jetem-8-1-s1:** 

*Basic metabolic panel (BMP)*	
Sodium	139 mEq/L
Chloride	100 mEq/L
Potassium	5.1 mEq/L
Bicarbonate (HCO_3_)	18 mEq/L
Blood Urea Nitrogen (BUN)	29 mg/dL
Creatine (Cr)	1.9 mg/dL
Glucose	176 mg/dL
Brain Natriuretic Peptide (BNP)	595 pg/mL (0–100 normal range)
High Sensitivity Troponin-1	55 ng/mL (4–60 normal range)

**Table t6-jetem-8-1-s1:** 

*Coagulation Studies*	
International Normalized Ratio	1.2
Prothrombin Time (PT)	26 seconds (10–14 normal range)
Partial Thromboplastin Time (aPTT)	33 seconds (24–38 normal range)

**Table t7-jetem-8-1-s1:** 

*Arterial Blood Gas (ABG)*	
pH	7.44
pCO2	38 mEq/L
paO2	60 mmHg
Bicarbonate (HCO_3_)	25 mEq/L

**Figure f6-jetem-8-1-s1:**
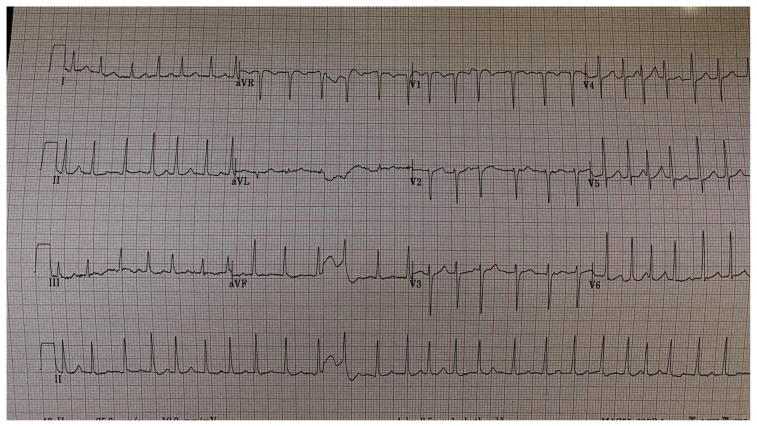
*Electrocardiogram (ECG) –* Atrial fibrillation with rapid ventricular response Image source: (author’s own image)

**Figure f7-jetem-8-1-s1:**
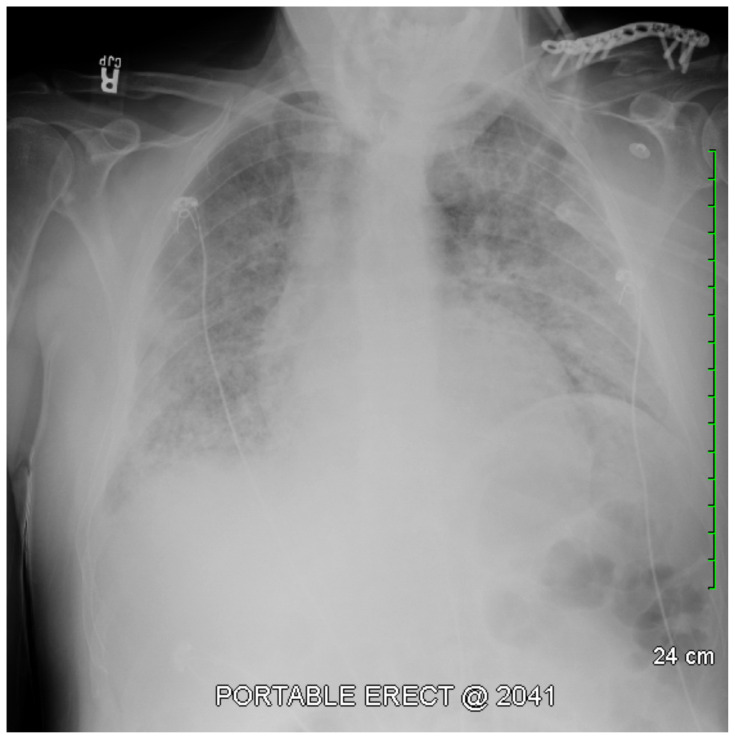
*Chest Radiograph (CXR) –* Pulmonary Edema Image source: Author’s own image

**Table t8-jetem-8-1-s1:** 

Minute (state)	Participant action/ trigger	Patient status (simulator response) & operator prompts	Monitor display (vital signs)
0:00 (Baseline)	Learner obtains history from the provider via tele-medicine. Learner should recognize severe respiratory distress during primary survey and request BiPAP or other forms of NIPPV.	Patient is in severe respiratory distress on 2L/min NC, diaphoretic, sitting upright in a stretcher, and speaking 2–3 word sentences when the case begins. History obtained from the provider via tele-medicine. If BiPAP/CPAP is not requested quickly, the patient will become unresponsive and more hypoxic requiring intubation.	T 99.0°F HR 132 BP 245/180 RR 32 O2 85% on 2L/min NC
BiPAP/CPAP Initiated	Learners should request BiPAP/CPAP. Learners should give specific settings and if not given, a confederate nurse will request them. Requests ECG and CXR. Requests labs.	If the patient is placed on BiPAP/CPAP, respirations and oxygen saturation should improve. If patient is placed on any other form of oxygen support, the patient will not improve until BiPAP, CPAP, or intubation is performed.	T 99.0°F HR 130 BP 235/170 RR 22 O2 94% on BiPAP O2 86% on NRB O2 85% on NC
Only use if BiPAP/CPAP not started within 10 minutes	If using as a telemedicine case, learner will instruct the confederate on how to perform the intubation. If using as a standard in-person case, the learner should perform an endotracheal intubation.	The patient’s respirations begin to slow down, and he becomes more hypoxic and unresponsive. The patient will now require endotracheal intubation. Vital signs will improve after intubation.	T 99.0°F HR 132 BP 245/180 RR 18 O2 70% Post-Intubation T 99.0°F HR 122 BP 190/100 RR 18 O2 94% ETT
Only use if the learner treats AFIB with RVR prior to BiPAP/CPAP and Nitroglycerin ECG shows Atrial Fibrillation with RVR	If the learner treats the AFIB prior to treating SCAPE, this branch point will start. Learner may likely use diltiazem, metoprolol, or amiodarone.	Patient’s ECG will show atrial fibrillation with RVR; however, the learner should recognize that this is due to SCAPE. If the learner attempts to treat the atrial fibrillation prior to administering BiPAP and/or Nitroglycerin, the patient should become more somnolent and hypoxic to the point of requiring intubation and mechanical ventilation. The HR and presence of atrial fibrillation with RVR should not improve with those medications. If the learner treats the patient appropriately with BiPAP and nitroglycerin, the RVR will improve.	T 99.0°F HR 130 BP 100/50 RR 30 O2 84% on NC or NRB O2 94% on BiPAP/CPAP
Nitroglycerin started	Nitroglycerin administered with BiPAP	Vitals will improve if the patient is given at least 400mcg IV bolus and/or infusion at 80mcg/min. Learner should then request patient to be transferred to their facility	T 99.0°F HR 95 BP 185/120 RR 22 O2 95% on BiPAP

**Table t9-jetem-8-1-s1:** 

**Critical Actions:** Request reassessment of the patient Request physical exam findings from the provider (only if using as a telemedicine case) Initiate non-invasive ventilation with BiPAP Administer nitrates Correctly interpret chest x-ray Request patient to be transferred once stabilized Interpret EKG as atrial fibrillation with rapid ventricular response	0:00

**Table t10-jetem-8-1-s1:** 

	Milestone	Did not achieve level 1	Level 1	Level 2	Level 3
1	**Emergency Stabilization (PC1)**	□ Did not achieve Level 1	□ Recognizes abnormal vital signs	□ Recognizes an unstable patient, requiring intervention Performs primary assessment Discerns data to formulate a diagnostic impression/plan	□ Manages and prioritizes critical actions in a critically ill patient Reassesses after implementing a stabilizing intervention
2	**Performance of focused history and physical (PC2)**	□ Did not achieve Level 1	□ Performs a reliable, comprehensive history and physical exam	□ Performs and communicates a focused history and physical exam based on chief complaint and urgent issues	□ Prioritizes essential components of history and physical exam given dynamic circumstances
3	**Diagnostic studies (PC3)**	□ Did not achieve Level 1	□ Determines the necessity of diagnostic studies	□ Orders appropriate diagnostic studies. Performs appropriate bedside diagnostic studies/procedures	□ Prioritizes essential testing Interprets results of diagnostic studies Reviews risks, benefits, contraindications, and alternatives to a diagnostic study or procedure
4	**Diagnosis (PC4)**	□ Did not achieve Level 1	□ Considers a list of potential diagnoses	□ Considers an appropriate list of potential diagnosis May or may not make correct diagnosis	□ Makes the appropriate diagnosis Considers other potential diagnoses, avoiding premature closure
5	**Pharmacotherapy (PC5)**	□ Did not achieve Level 1	□ Asks patient for drug allergies	□ Selects an medication for therapeutic intervention, consider potential adverse effects	□ Selects the most appropriate medication and understands mechanism of action, effect, and potential side effects Considers and recognizes drug-drug interactions
6	**Observation and reassessment (PC6)**	Did not achieve Level 1	Reevaluates patient at least one time during case	Reevaluates patient after most therapeutic interventions	Consistently evaluates the effectiveness of therapies at appropriate intervals
7	**Disposition (PC7)**	□ Did not achieve Level 1	□ Appropriately selects whether to admit or discharge the patient	□ Appropriately selects whether to admit or discharge Involves the expertise of some of the appropriate specialists	□ Educates the patient appropriately about their disposition Assigns patient to an appropriate level of care (ICU/Tele/Floor) Involves expertise of all appropriate specialists
9	**General Approach to Procedures (PC9)**	Did not achieve Level 1	Identifies pertinent anatomy and physiology for a procedure Uses appropriate Universal Precautions	Obtains informed consent Knows indications, contraindications, anatomic landmarks, equipment, anesthetic and procedural technique, and potential complications for common ED procedures	Determines a back-up strategy if initial attempts are unsuccessful Correctly interprets results of diagnostic procedure
20	**Professional Values (PROF1)**	□ Did not achieve Level 1	□ Demonstrates caring, honest behavior	□ Exhibits compassion, respect, sensitivity and responsiveness	□ Develops alternative care plans when patients’ personal beliefs and decisions preclude standard care
22	**Patient centered communication (ICS1)**	□ Did not achieve level 1	□ Establishes rapport and demonstrates empathy to patient (and family) Listens effectively	□ Elicits patient’s reason for seeking health care	□ Manages patient expectations in a manner that minimizes potential for stress, conflict, and misunderstanding. Effectively communicates with vulnerable populations, (at risk patients and families)
23	**Team management (ICS2)**	□ Did not achieve level 1	□ Recognizes other members of the patient care team during case (nurse, techs)	□ Communicates pertinent information to other healthcare colleagues	□ Communicates a clear, succinct, and appropriate handoff with specialists and other colleagues Communicates effectively with ancillary staff

## INSTRUCTOR MATERIALS

**Case Title:** Telemedicine Consult for Shortness of Breath Due to Sympathetic Crashing Acute Pulmonary Edema

**Case Description & Diagnosis (short synopsis):** A 59-year-old male presents to a critical access hospital due to shortness of breath. He has a history of insulin dependent diabetes, atrial fibrillation, and hypertension. A nurse practitioner at the critical access hospital has initiated a telemedicine consult that the learner will conduct via teleconferencing video call. The patient will currently be hypoxic despite being on a nonrebreather for the past 10 minutes. The confederate practitioner will discuss the case with the learner and ask for additional help in management. The confederate should tell the learner the entire history and physical exam as well as treatments that have already been attempted. The learner should recognize that the patient is in sympathetic crashing acute pulmonary edema (SCAPE) and request that he be placed on BiPAP or CPAP and be given nitroglycerin bolus(es) and/or infusion. At the conclusion of the case, the patient should be transferred. This case can also be performed without the telemedicine component.

**Equipment or Props Needed:**

High-fidelity human mannequin

Nasal cannula

Non-rebreather mask

Bilateral positive airway pressure (BiPAP) or continuous positive airway pressure (CPAP) device IV catheters 18 or 20 gauges

Cardiac monitor

Pulse oximetry

Intravenous (IV) pole

Defibrillator Cart

Endotracheal intubation equipment (laryngoscope handle, Miller/Macintosh blades, endotracheal tube, stylet, 10mL syringe, and self-inflating bag)

Telemedicine Component:

– Laptop or tablet with video conferencing capabilities
– Table or cart for the laptop

**Confederates needed:**

Advanced practice provider at critical access hospital

**Stimulus Inventory:[Table t2-jetem-8-1-s1]**

**Background and brief information:** 59-year-old male presents via private vehicle to a critical access hospital due to worsening shortness of breath. He has a history of insulin dependent diabetes, atrial fibrillation, and hypertension.

**Initial presentation:** 59-year-old male who presents with shortness of breath and appears to be in severe respiratory distress.

**How the scene unfolds:** The learner(s) is/are consulted via tele-medicine from a critical access hospital regarding a patient in severe respiratory distress due to SCAPE. The learners should ask the confederates about their initial assessment, interventions, and changes in the patient’s initial presentation. The patient will have a nasal cannula at 2L/min; however, symptoms and vitals on presentation will be unchanged, which prompted the tele-medicine consult. Based on the patient’s presentation, vitals, and physical exam findings, the learner(s) should suspect SCAPE and request an electrocardiogram, chest x-ray, BiPAP, and nitrates. If any other non-positive pressure devices are used, the patient will not improve. If BiPAP and nitrates are not given within 5 minutes, the patient will become unresponsive, develop worsening hypoxia, and require intubation with mechanical ventilation. If appropriate medications and BiPAP are initiated in a timely manner, the patient will improve. The patient’s initial electrocardiogram will be atrial fibrillation with rapid ventricular response (AFIB with RVR). The learner should recognize that the patient’s underlying diagnosis (SCAPE) is the reason for AFIB with RVR and should stabilize the patient before treating the arrhythmia. If the arrhythmia is treated before stabilization, the patient’s condition will further deteriorate at the discretion of the instructor until the pulmonary edema is treated.

**Critical actions:**

1. Request reassessment of the patient
2. Request physical exam findings from the provider (only if using as a telemedicine case)
3. Initiate non-invasive ventilation with BiPAP or CPAP
4. Administer nitrates
5. Correctly interpret chest X-ray
6. Request patient to be transferred once stabilized (only if using as a telemedicine case)
7. Interpret EKG as atrial fibrillation with rapid ventricular response

**Case Title:** Telemedicine Consult for Shortness of Breath Due to Sympathetic Crashing Acute Pulmonary Edema

**Chief Complaint:** 59-year-old male with a history of congestive heart failure (CHF), hypertension (HTN), insulin dependent diabetes mellitus (IDDM), and atrial fibrillation (AFIB) presenting with sudden onset shortness of breath for the past 2–3 hours[Table t3-jetem-8-1-s1]

**General Appearance:** Alert, Sitting upright on stretcher, Diaphoretic, Ill-appearing, Severe respiratory distress

**Primary Survey:**

- **Airway:** Patent
- **Breathing:** Tachypneic, Speaks in 2–3 word sentences, Diffuse rales bilaterally
- **Circulation:** Tachycardic, Hypertensive, Symmetrical peripheral pulses

**History:**

- **History of present illness:** 59-year-old with worsening sudden onset shortness of breath over the past 2–3 hours.
- Past medical history: HTN, IDDM, and AFIB
- Past surgical history: Appendectomy
- **Patient’s medications:** Metoprolol, Rivaroxiban, Novolog, Levemir, and Lisinopril
- Allergies: None
- **Social history:** Lives with wife, nonsmoker, no alcohol use, no recreational drug use
- **Family history:** Unknown because patient was adopted

**Secondary Survey/Physical Examination:**

- **General appearance:** Severe respiratory distress, Diaphoretic, Sitting upright on stretcher
- HEENT:
  ○ **Head:** Within normal limits
  ○ **Eyes:** Within normal limits
  ○ **Ears:** Within normal limits
  ○ **Nose:** Within normal limits
  ○ **Throat:** Within normal limits
- **Neck:** Jugular Venous Distention, Trachea midline
- **Heart:** Tachcyardic, diffuse systolic ejection murmur
- **Lungs:** Severe respiratory distress, Diffuse rales bilaterally, speaks in 2–3 word sentences
- **Abdominal/GI:** Soft, Nontender, No distention
- **Extremities:** 1+ pitting edema in the lower extremities, non tender
- **Neuro:** Alert, Answers questions appropriately
- **Skin:** Warm, dry, and intact

**Results:** [Table t4-jetem-8-1-s1] [Table t5-jetem-8-1-s1] [Table t6-jetem-8-1-s1] [Table t7-jetem-8-1-s1]

## OPERATOR MATERIALS

**SIMULATION EVENTS TABLE:** [Table t8-jetem-8-1-s1]

**Diagnosis:**

Sympathetic crashing acute pulmonary edema

**Disposition:**

Transfer is using as a tele-medicine case

Intensive care unit if not using as a tele-medicine case

## DEBRIEFING AND EVALUATION PEARLS

### Sympathetic Crashing Acute Pulmonary Edema

#### Reaction phase questions

1. How well was your recognition and treatment of this patient?
2. Is there anything you would have done differently?
3. How was your communication affected by using the tele-medicine medium?
4. How was your ability to assess and treat the patient affected by using the tele-medicine medium?
5. Discuss how it felt having to rely on someone else’s physical exam and assessment in order to treat this patient

#### Understanding phase questions

1. Since SCAPE is within the spectrum of acute heart failure syndromes, how were you able to differentiate this from fluid overload pulmonary edema, cardiogenic shock, or congestive heart failure exacerbation?
2. Discussion regarding the presence of atrial fibrillation:
  - If the learner decided to treat AF with RVR directly, ask them their reasoning behind this
  - If the learner chose to treat the underlying cause (SCAPE) of AF with RVR, ask them their reasoning

### Sympathetic Crashing Acute Pulmonary Edema

#### Pathophysiology

Typically occurs in the setting of uncontrolled or poorly controlled hypertension. It is a type of hypertensive emergency and also within the spectrum of acute heart failure syndromes (AHFS). While other AHFS develop over days to weeks, SCAPE develops within minutes to hours. The elevated systemic blood pressure leads to an increase in afterload. In part, this develops into poor peripheral perfusion and pulmonary edema. Due to decreased perfusion, there is a reflexive release of sympathetic mediators. Unfortunately, this sympathetic surge leads to peripheral vasoconstriction and pulmonary vasodilation, which further worsens afterload and pulmonary edema. Unlike other conditions associated with acute heart failure, patients with SCAPE are typically euvolemic or hypovolemic; therefore, diuretics typically will not improve this condition and have the potential to make it worse. Overall, the increased afterload leads to pulmonary edema as blood cannot efficiently leave the left side of the heart. This pulmonary edema makes the patient feel more dyspneic leading to further distress and more sympathetic activation. The repeated sympathetic activation causes additional vasoconstriction, particularly in the splanchnic vascular system. Pulmonary edema will once again further worsen due to this intravascular fluid shift from peripheral vasoconstriction and pulmonary vasodilation.

#### Diagnosis

Because SCAPE must be recognized and treated rapidly, it is typically a clinical diagnosis. It should be recognized by rapid onset dyspnea over minutes to hours, systolic blood pressure greater than 180mmHg, and clinical and/or radiographic evidence of pulmonary edema. There are no specific laboratory values that confirm the diagnosis of SCAPE, although typically the BNP or pro-BNP will be elevated.

#### Treatments

Despite the initial severity of SCAPE, invasive ventilation can often be avoided with rapid recognition and treatment with noninvasive ventilation (NIV) and systemic nitrates. Two types of NIV are bi-level positive airway pressure (BiPAP) and continuous positive airway pressure (CPAP). Noninvasive ventilation is able to utilize pulmonary dead space while reducing the preload and afterload by increasing intrathoracic pressure. Nitrates provide further benefit by also reducing the preload and afterload. Lower doses of nitrates will only provide venodilation, but as dosage is increased, they will also cause arteriodilation. If IV access is not immediately available, sublingual and topical nitrates can be utilized. Once IV access is obtained, boluses of nitroglycerin can be given, typically 400–800 mcg every 5 minutes. This is then followed by a nitroglycerin infusion at 80–100 ug/min and then titrated to the desired effect. If recognized and treated rapidly, patients can be weaned off the infusion and BiPAP while in the emergency department. Sublingual captopril or IV enalapril can also be used to provide further afterload reduction as needed. Furosemide has shown poor evidence in improving SCAPE. In this subset of patients, it can have a negative effect because it activates the renin-angiotensin-aldosterone-system (RAAS) and sympathetic nervous system.

### Atrial Fibrillation

Atrial fibrillation (AFIB) is the most commonly treated cardiac arrhythmia and characterized as an irregularly irregular ventricular rhythm and absence of P waves. It can be further classified as paroxysmal, persistent, long-standing persistent, and permanent. Presentations can vary from asymptomatic to palpitations, dizziness, syncope, nausea, and/or chest pain among many others. Treatment of AFIB in the emergency department is dependent on hemodynamic stability and risk stratification. In the hemodynamically unstable patient, synchronized cardioversion is the preferred treatment. Cardioversion without anticoagulation can also be performed if the arrhythmia has been present for less than 48 hours. Stable patients can initially be treated with intravenous rate control agents such metoprolol (lopressor) or diltiazem (cardizem). Metoprolol dosing is given as 5mg every two to five minutes for a maximum of 3 doses. If this is effective in reducing the heart rate to below 110 beats per minute (bpm), the patient is given oral metoprolol tartrate 25–100mg. Diltiazem is another first line option and is dosed at 0.25mg/kg IV. A second dose at 0.35mg/kg can be given if the first attempt does not reduce the heart rate to below 110 bpm. If both of these doses fail to achieve rate control, a diltiazem infusion ranging from 5–15mg/hr can be started.

Determining if there is an underlying cause such as SCAPE, pulmonary embolism, or myocardial infarction should also weighin on how AFIB is treated. Typically, as in this simulation, treatment of the underlying cause is ideal. In some studies, attempts at rate and/or rhythm control of AFIB when the patient has an acute medical illness may lead to a higher risk of adverse effects.^7^

### Telemedicine

Telemedicine is the process of delivering health services and information through an electronic means ranging from video conferencing to messaging.^8^ It can allow board certified emergency medicine physicians to assist in providing care in rural emergency departments and smaller hospitals who may be staffed with less experienced emergency medicine providers. In order to provide appropriate patient care, telemedicine must provide information that is equal or superior to an in-person examination. The telemedicine visit must also conform to the standards of medical care that are expected of an in-person visit and incorporate diagnostic testing if needed to reach an accurate diagnosis.^9^ We believe it is important to familiarize our residents with telemedicine since it may become a larger part of the practice of emergency medicine in the future.

## SIMULATION ASSESSMENT

*Telemedicine Consult for Shortness of Breath Due to Sympathetic Crashing Acute Pulmonary Edema*

Learner: _________________________________________

***Assessment Timeline***

This timeline is to help observers assess their learners. It allows observer to make notes on when learners performed various tasks, which can help guide debriefing discussion.[Table t9-jetem-8-1-s1]

**Critical Actions:**

□ Request reassessment of the patient
□ Request physical exam findings from the provider (only if using as a telemedicine case)
□ Initiate non-invasive ventilation with BiPAP
□ Administer nitrates
□ Correctly interpret chest x-ray
□ Request patient to be transferred once stabilized
□ Interpret EKG as atrial fibrillation with rapid ventricular response

**Summative and formative comments:**

**Milestones assessment:** [Table t10-jetem-8-1-s1]
