# Supplementary material for: Telemedicine Consult for Shortness of Breath Due to Sympathetic Crashing Acute Pulmonary Edema
Source: J Educ Teach Emerg Med. 2023 Jan 31;8(1):S1–S24. doi: 10.21980/J8HS86 (PMC10332770; doi:10.21980/J8HS86)
Supplement: Supplementary file 1 [file jetem-8-1-S1-supp1.pptx]

## Slide 1
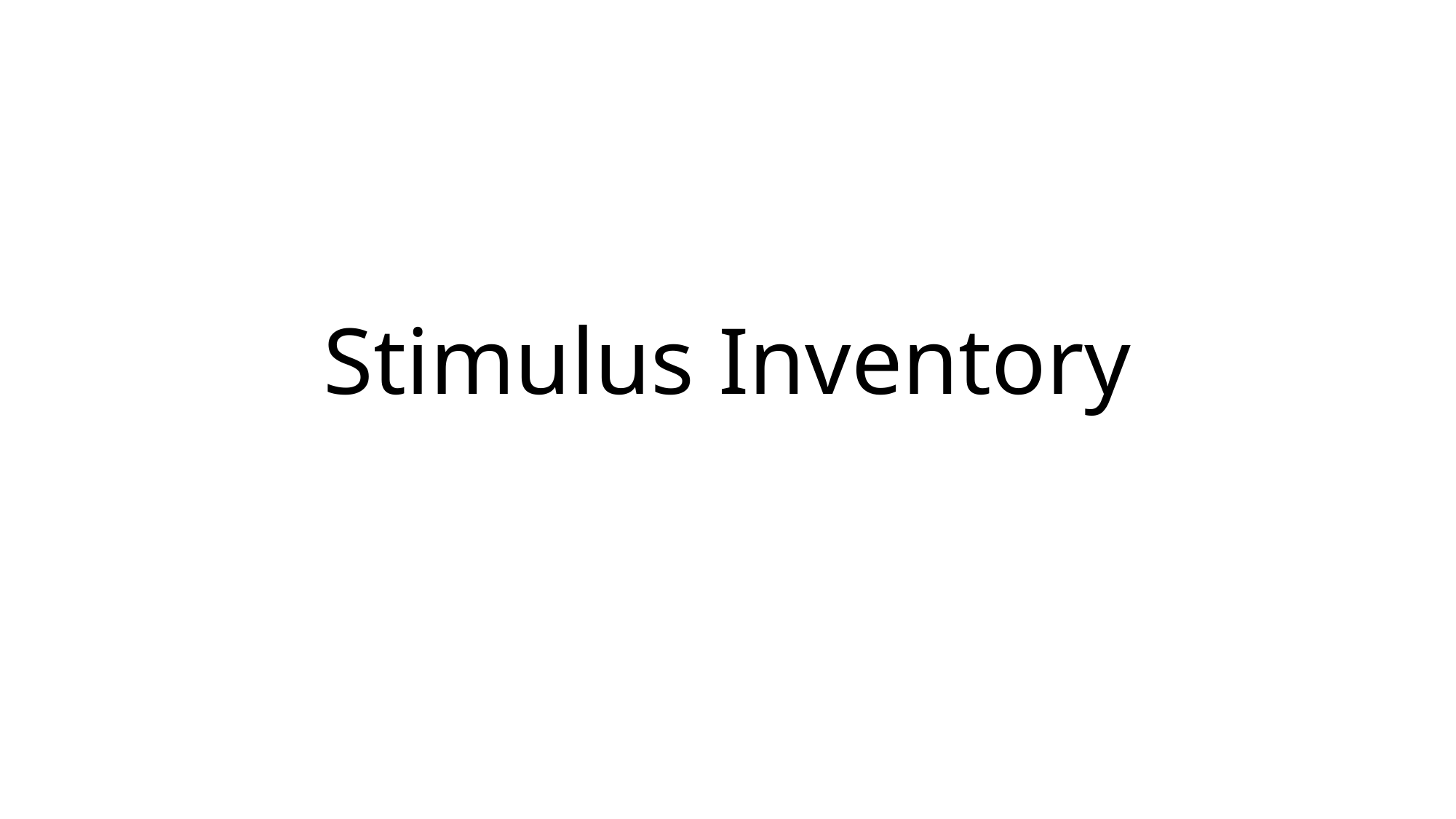

# Stimulus Inventory

## Slide 2
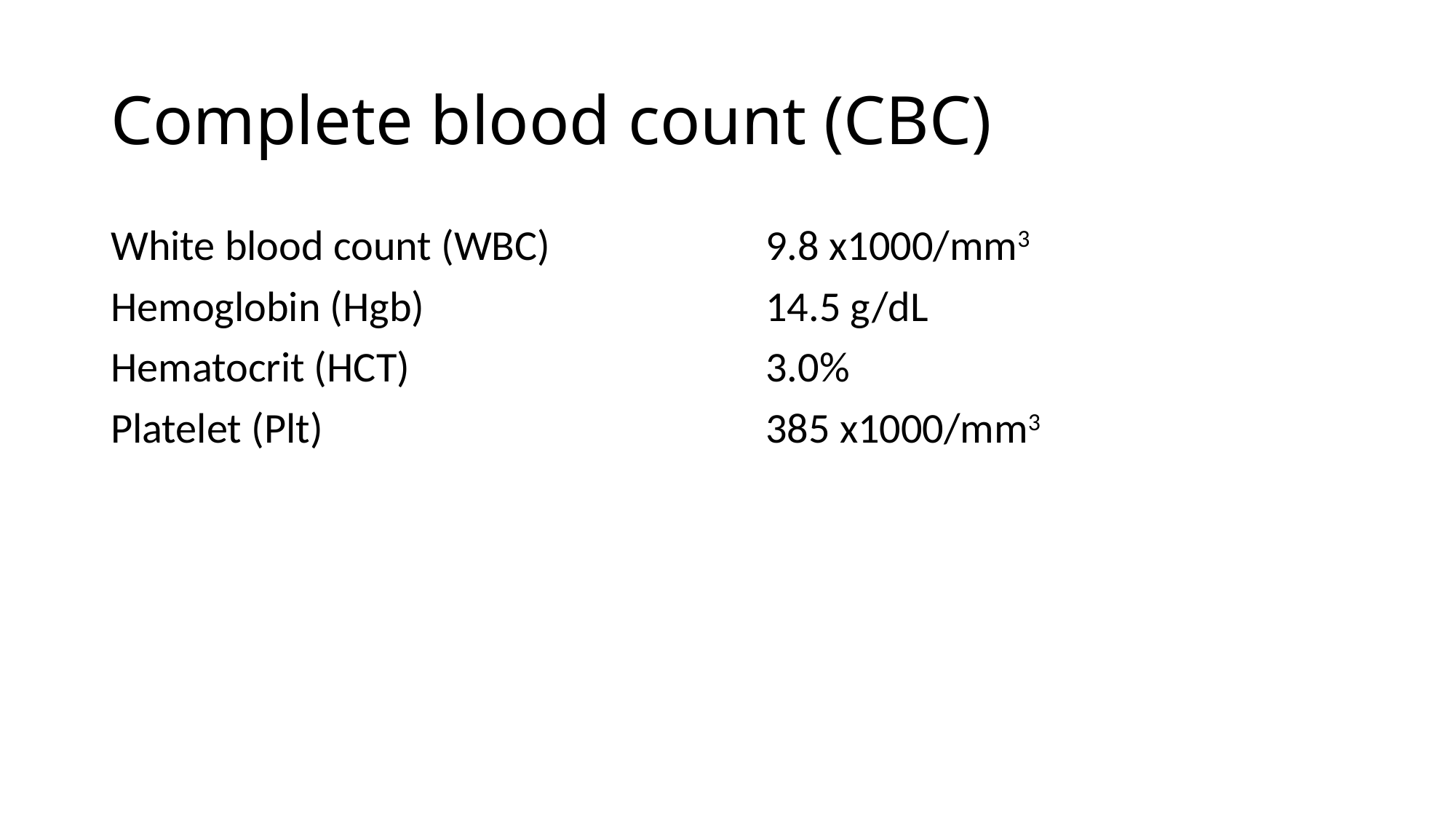

# Complete blood count (CBC)
White blood count (WBC) 		9.8 x1000/mm3
Hemoglobin (Hgb)				14.5 g/dL
Hematocrit (HCT)				3.0%
Platelet (Plt) 				385 x1000/mm3

## Slide 3
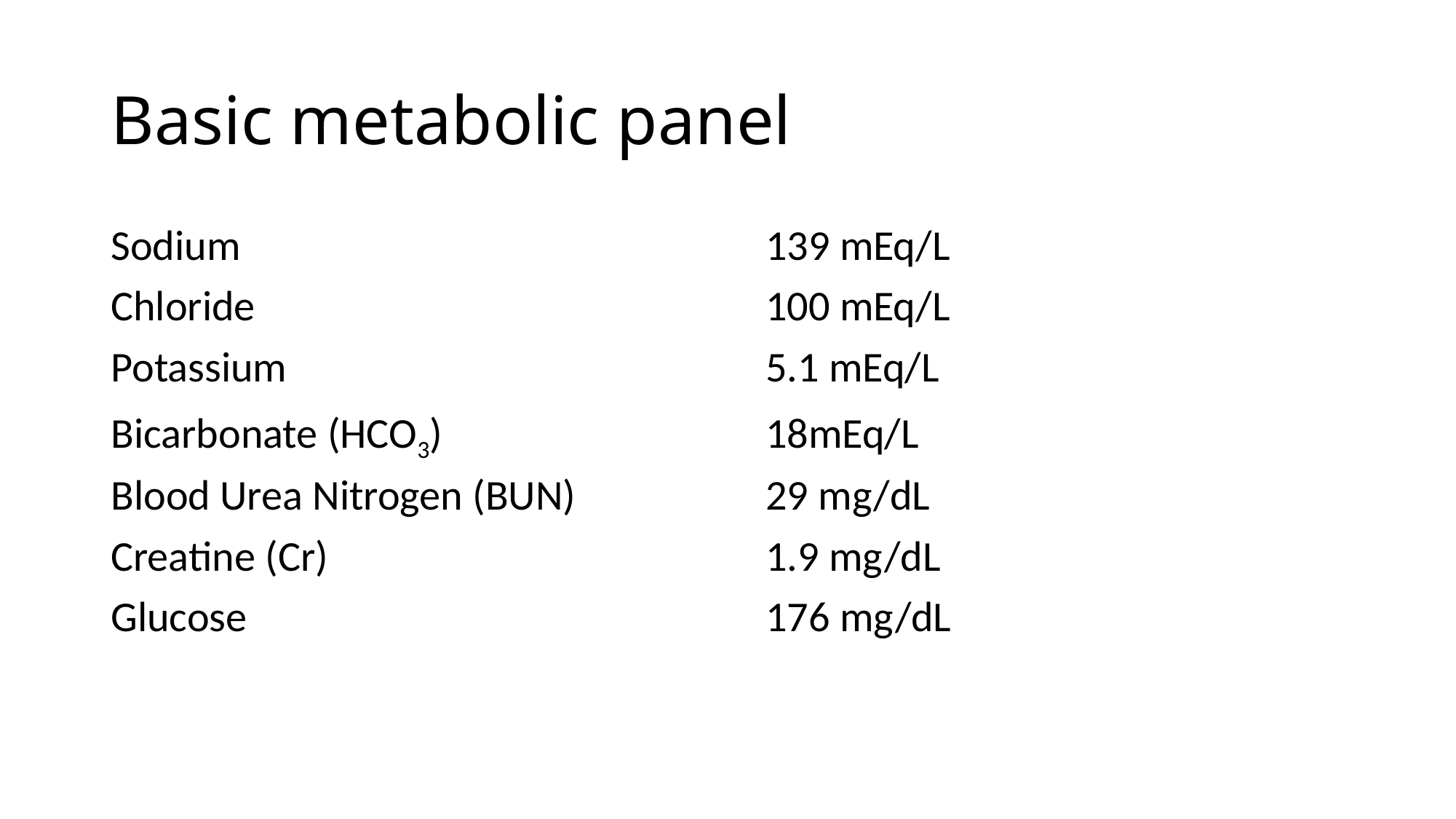

# Basic metabolic panel
Sodium 					139 mEq/L
Chloride 					100 mEq/L
Potassium					5.1 mEq/L
Bicarbonate (HCO3)			18mEq/L
Blood Urea Nitrogen (BUN)		29 mg/dL
Creatine (Cr)		 			1.9 mg/dL
Glucose 					176 mg/dL

## Slide 4
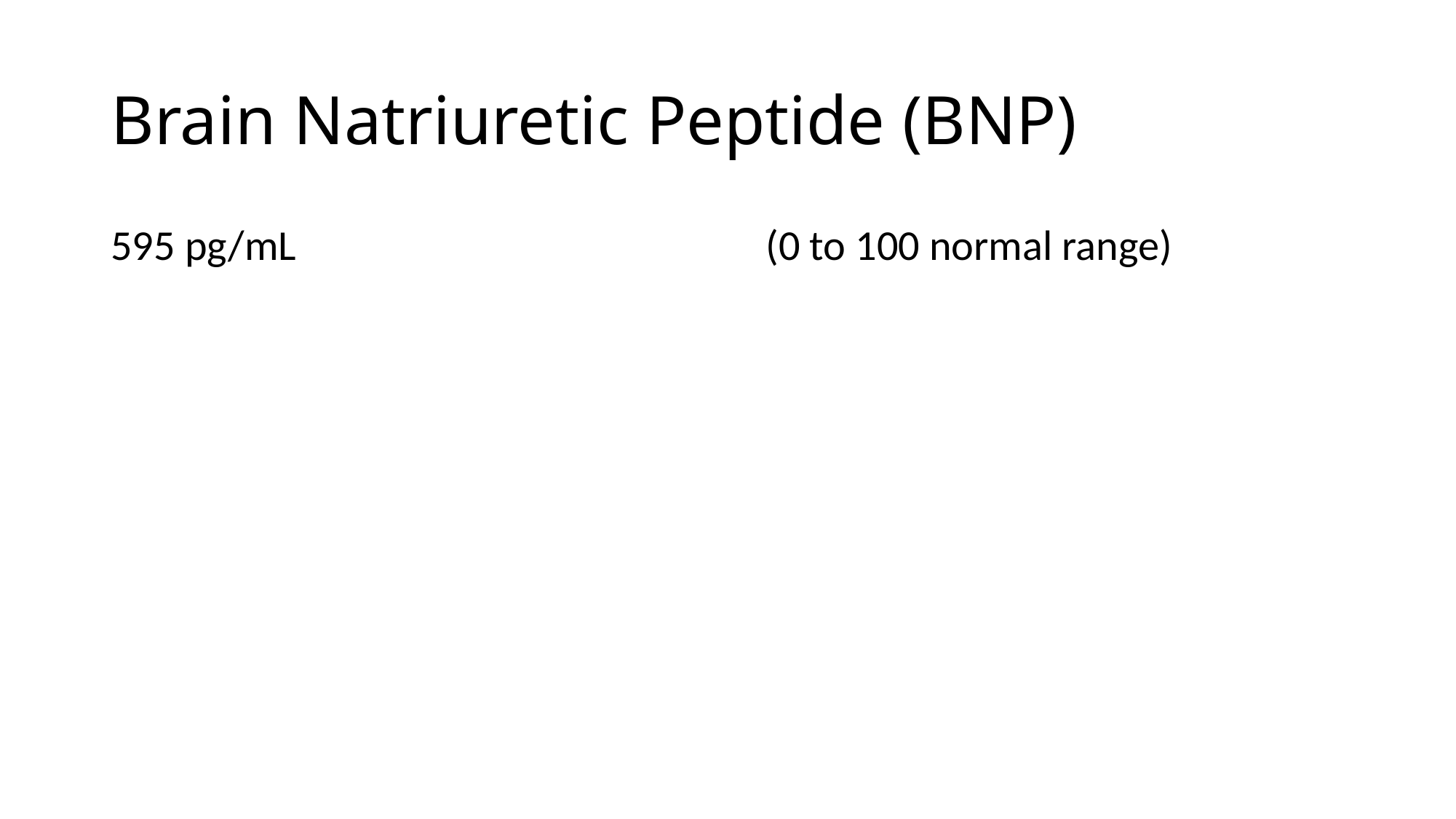

# Brain Natriuretic Peptide (BNP)
595 pg/mL					(0 to 100 normal range)

## Slide 5
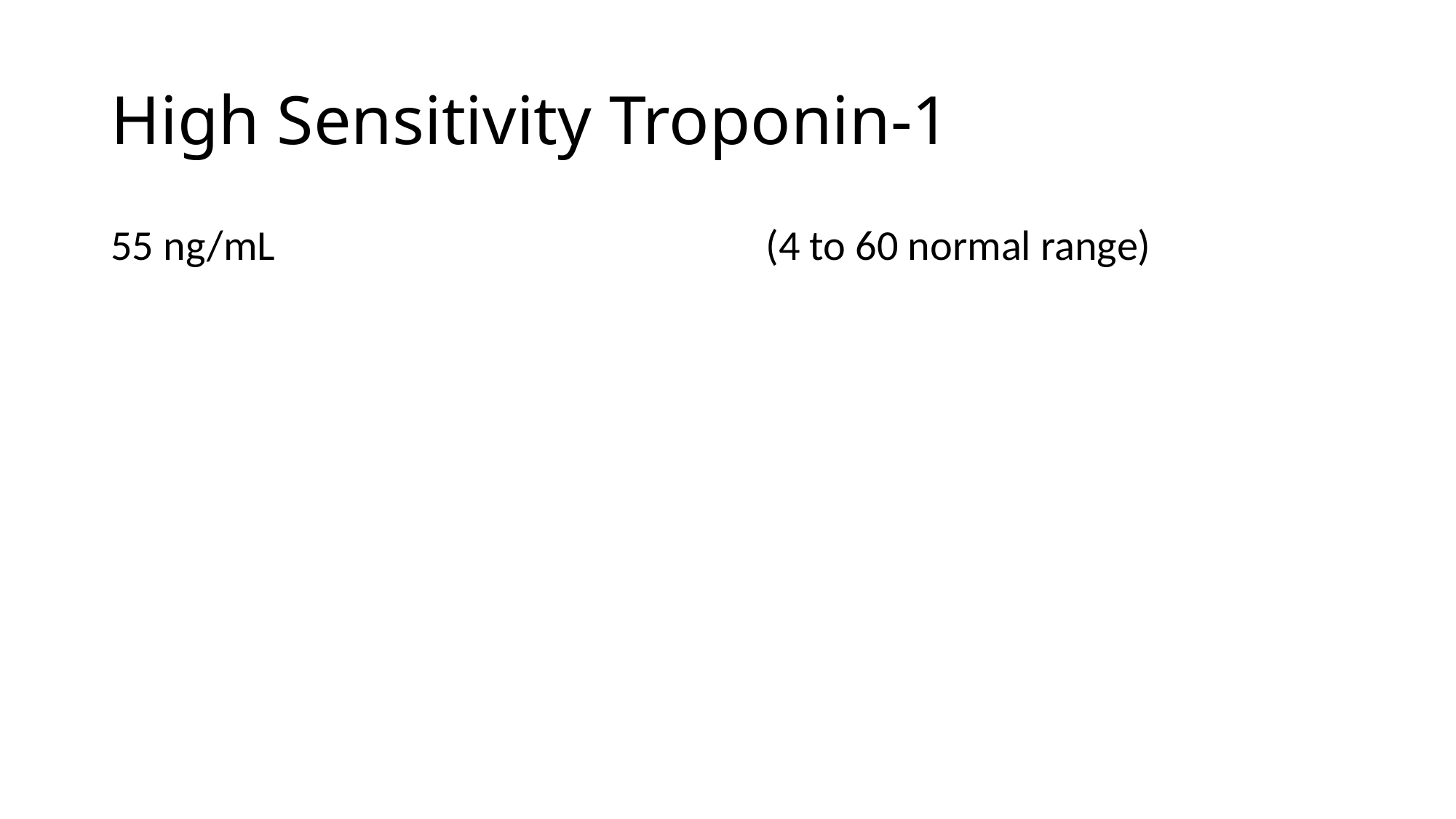

# High Sensitivity Troponin-1
55 ng/mL					(4 to 60 normal range)

## Slide 6
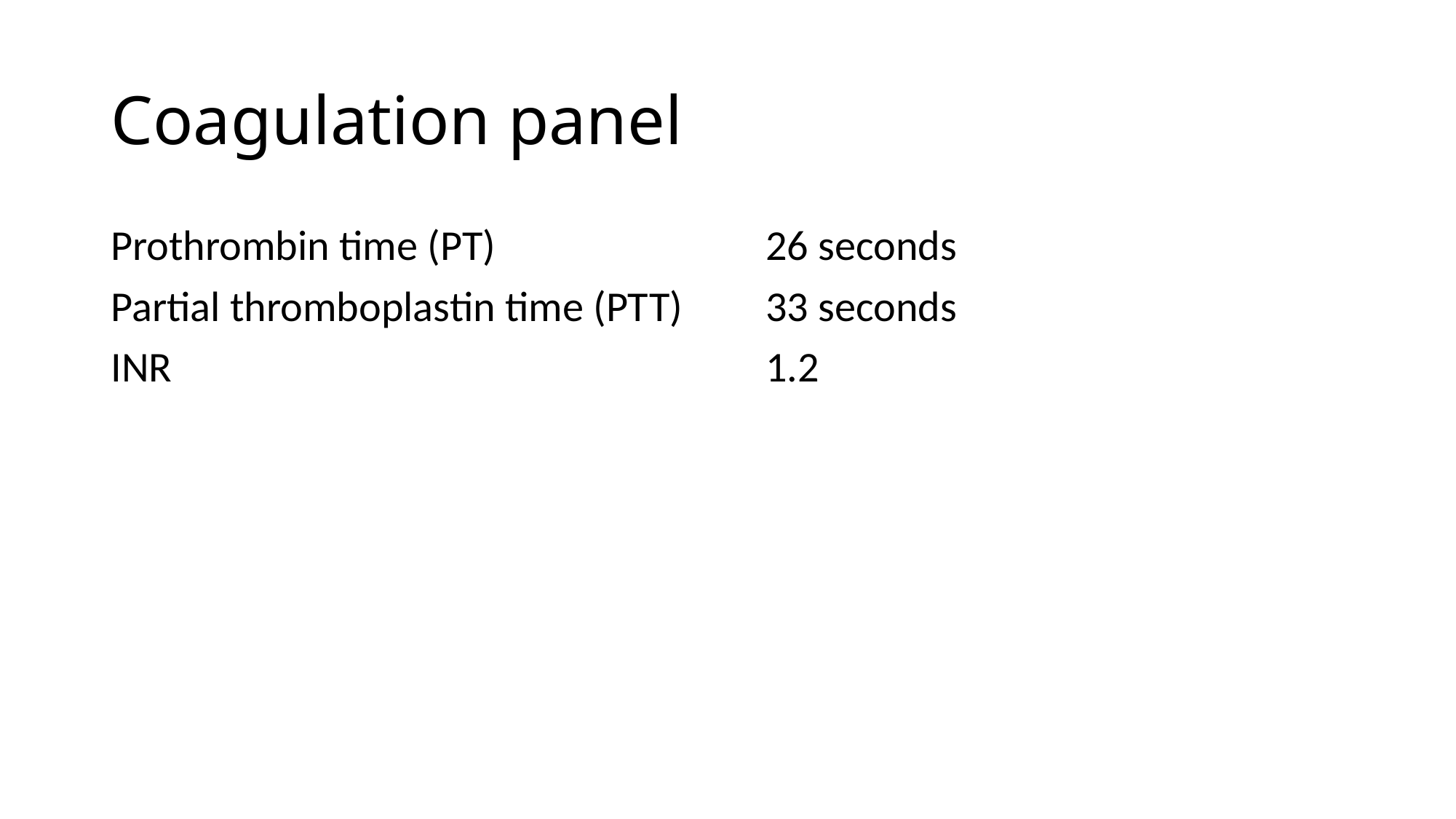

# Coagulation panel
Prothrombin time (PT) 			26 seconds
Partial thromboplastin time (PTT) 	33 seconds
INR 						1.2

## Slide 7
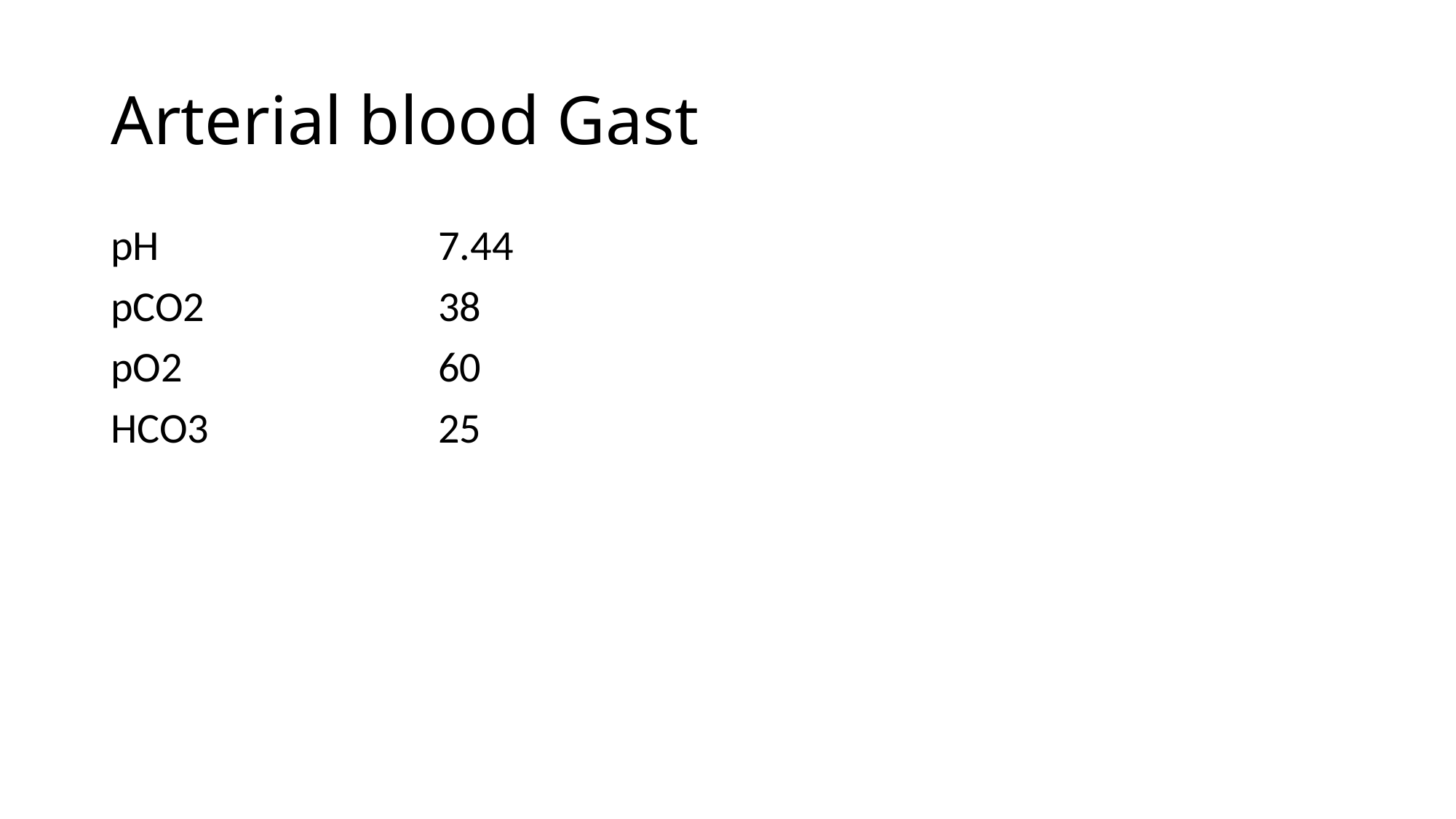

# Arterial blood Gast
pH			7.44
pCO2			38
pO2			60
HCO3			25

## Slide 8
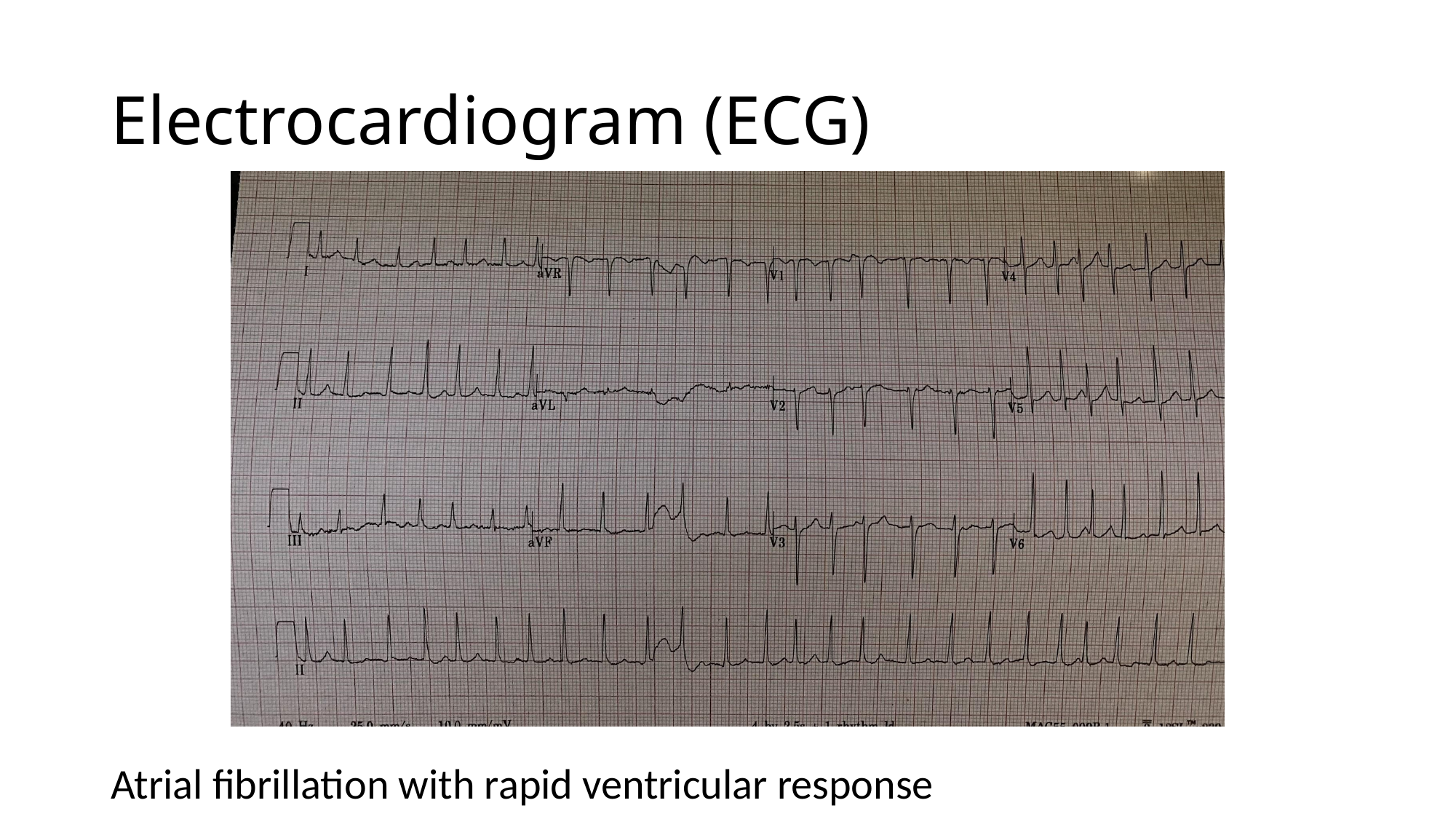

# Electrocardiogram (ECG)
Atrial fibrillation with rapid ventricular response

## Slide 9
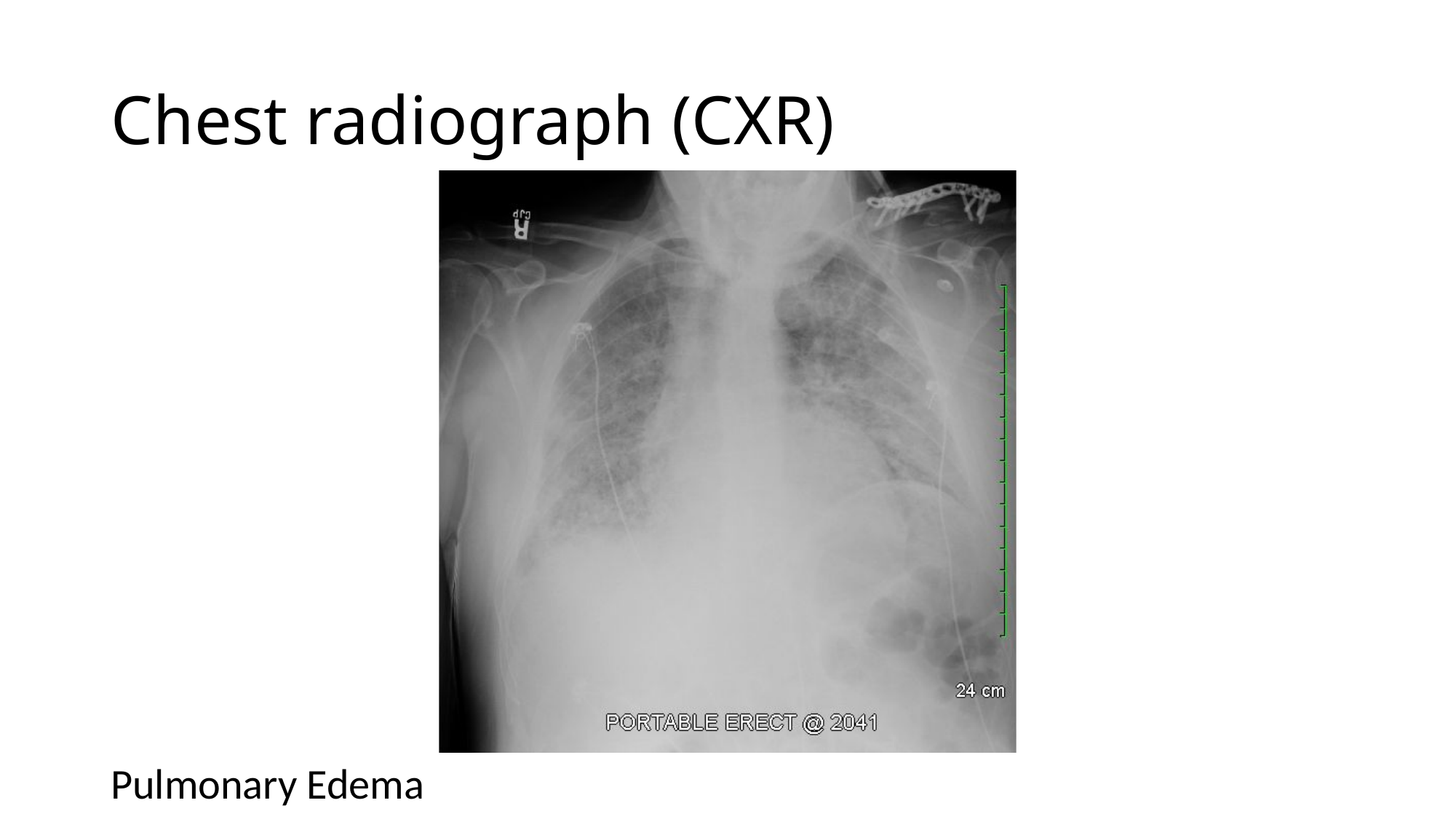

# Chest radiograph (CXR)
Pulmonary Edema
