# Supplementary figures and images for: Telemedicine Consult for Shortness of Breath Due to Sympathetic Crashing Acute Pulmonary Edema
Source: J Educ Teach Emerg Med. 2023 Jan 31;8(1):S1–S24. doi: 10.21980/J8HS86 (PMC10332770; doi:10.21980/J8HS86)

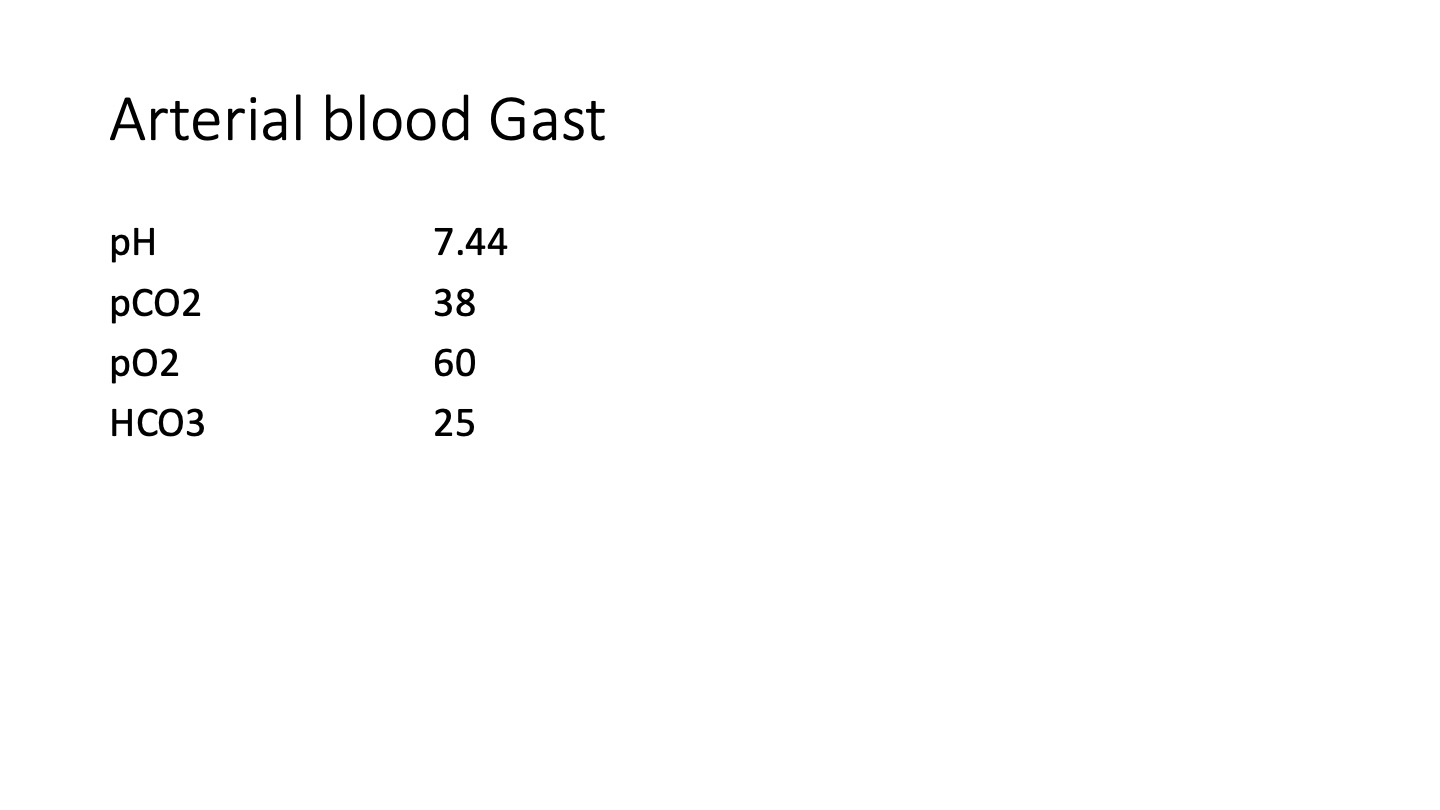

Supplement: Supplementary file 2 [file jetem-8-1-S1-supp2.jpg]

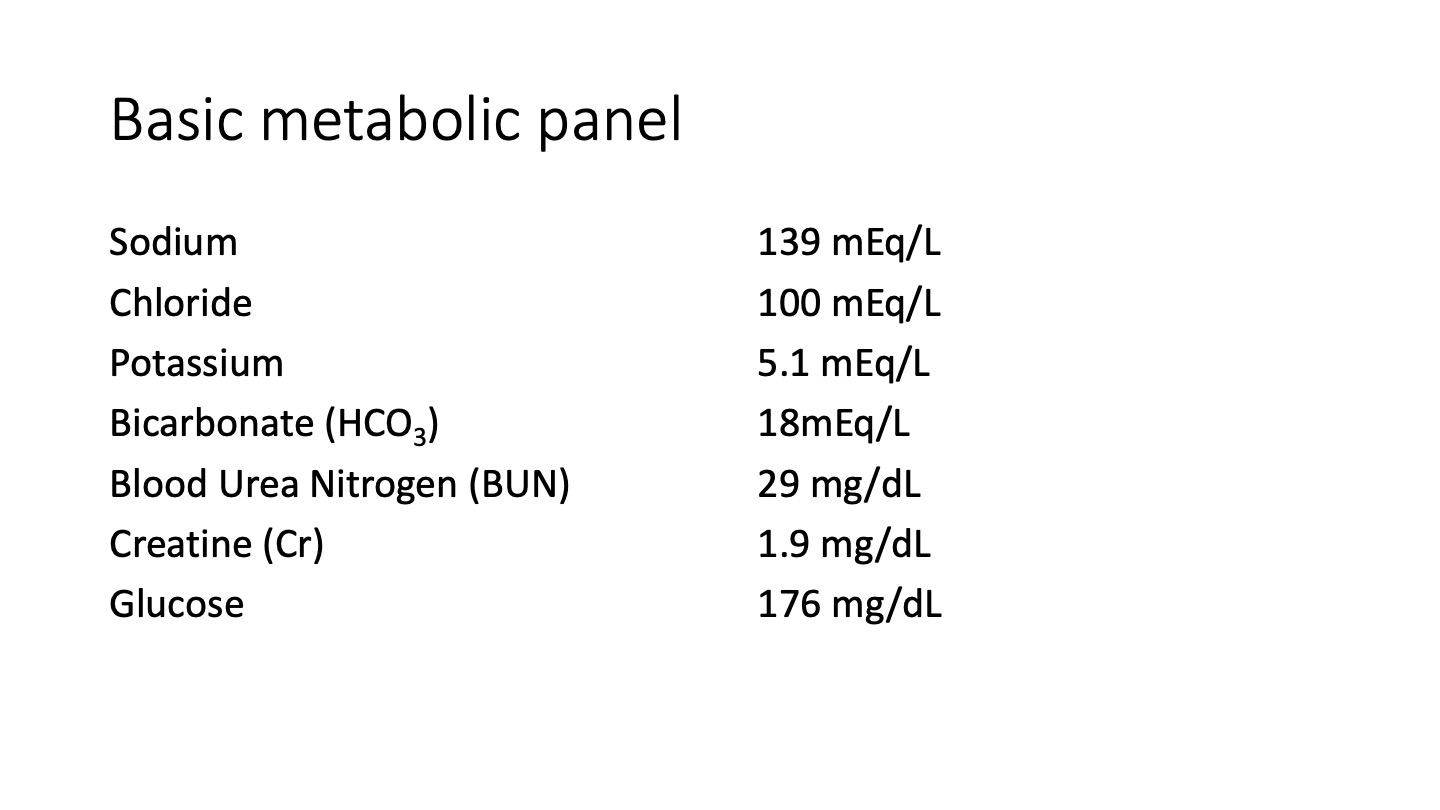

Supplement: Supplementary file 3 [file jetem-8-1-S1-supp3.jpg]

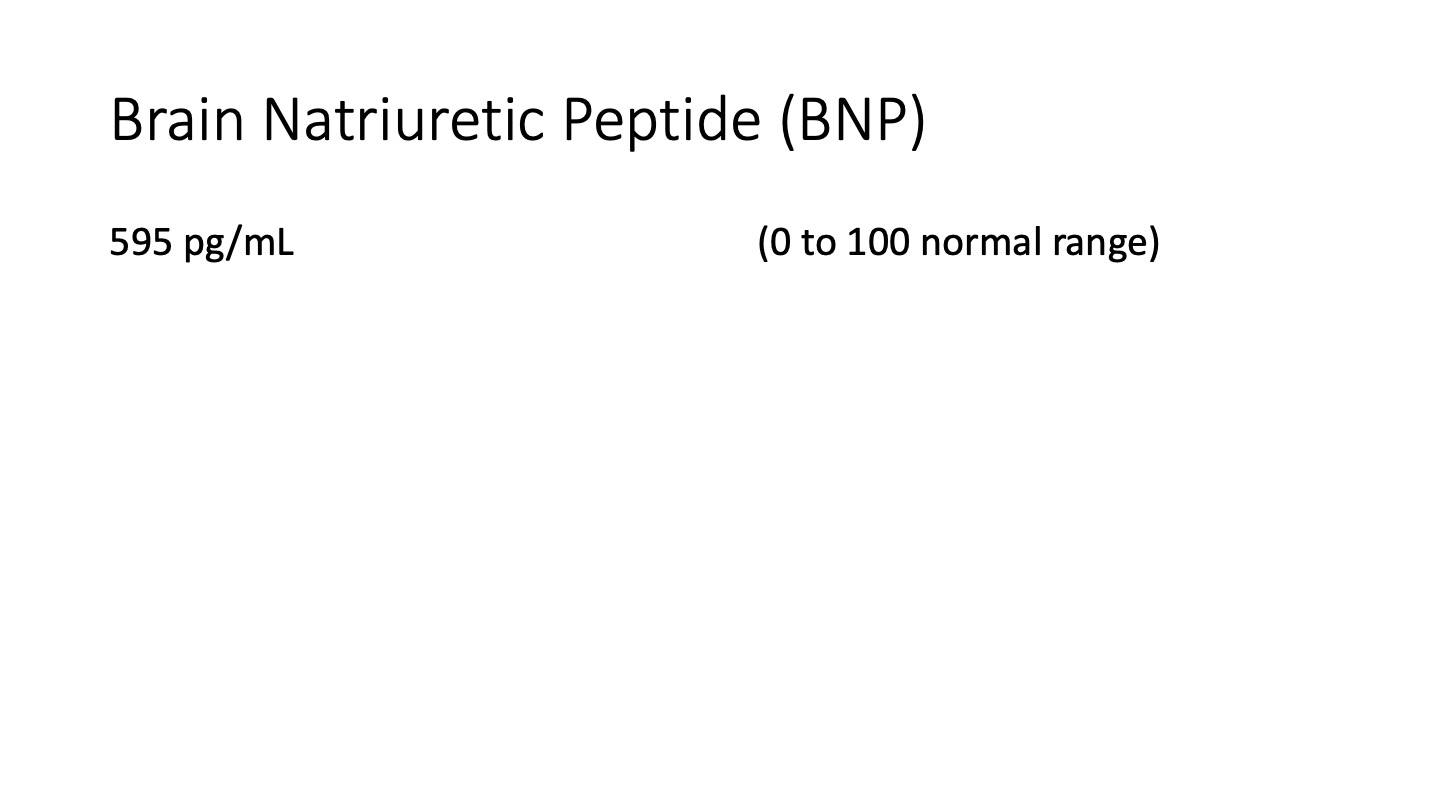

Supplement: Supplementary file 4 [file jetem-8-1-S1-supp4.jpg]

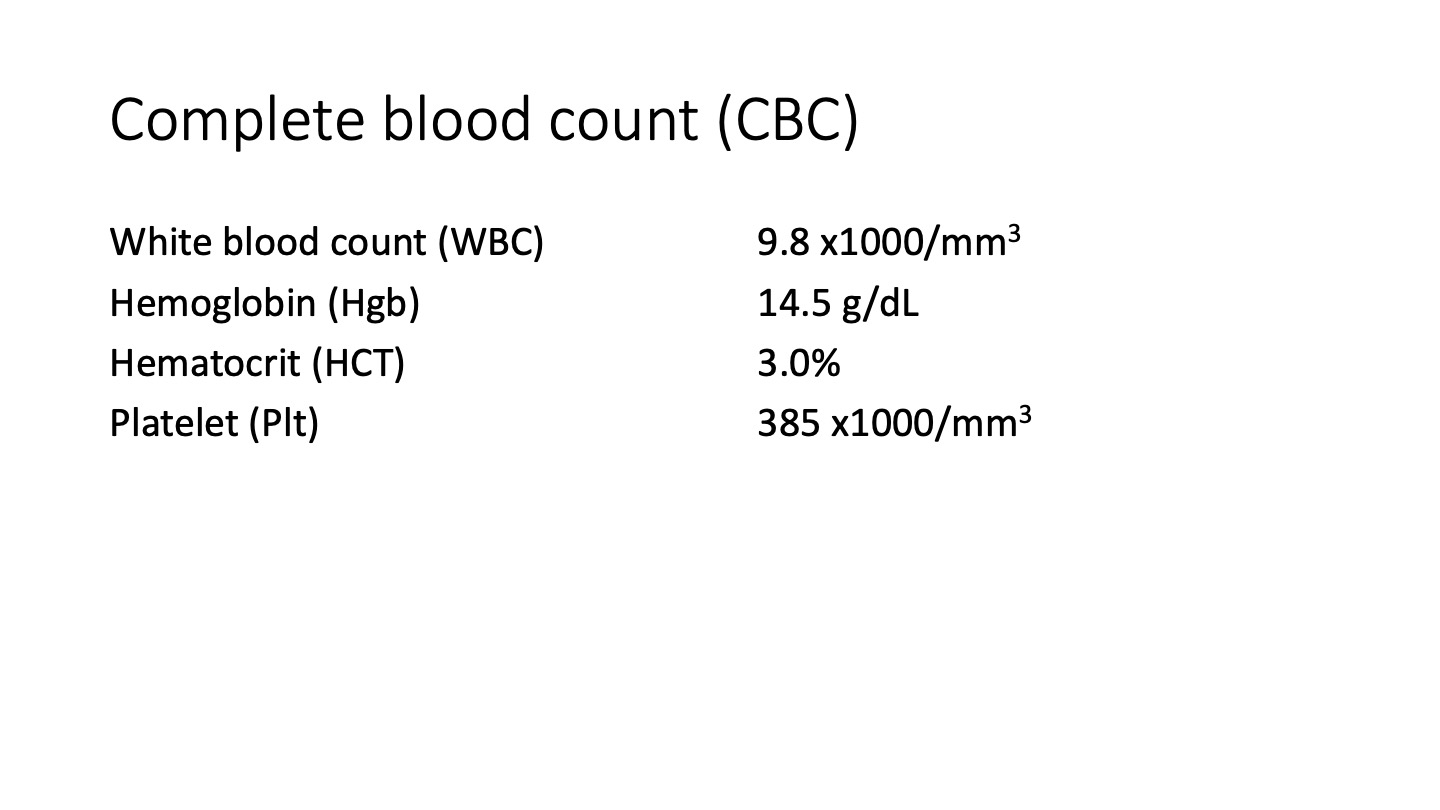

Supplement: Supplementary file 5 [file jetem-8-1-S1-supp5.jpg]

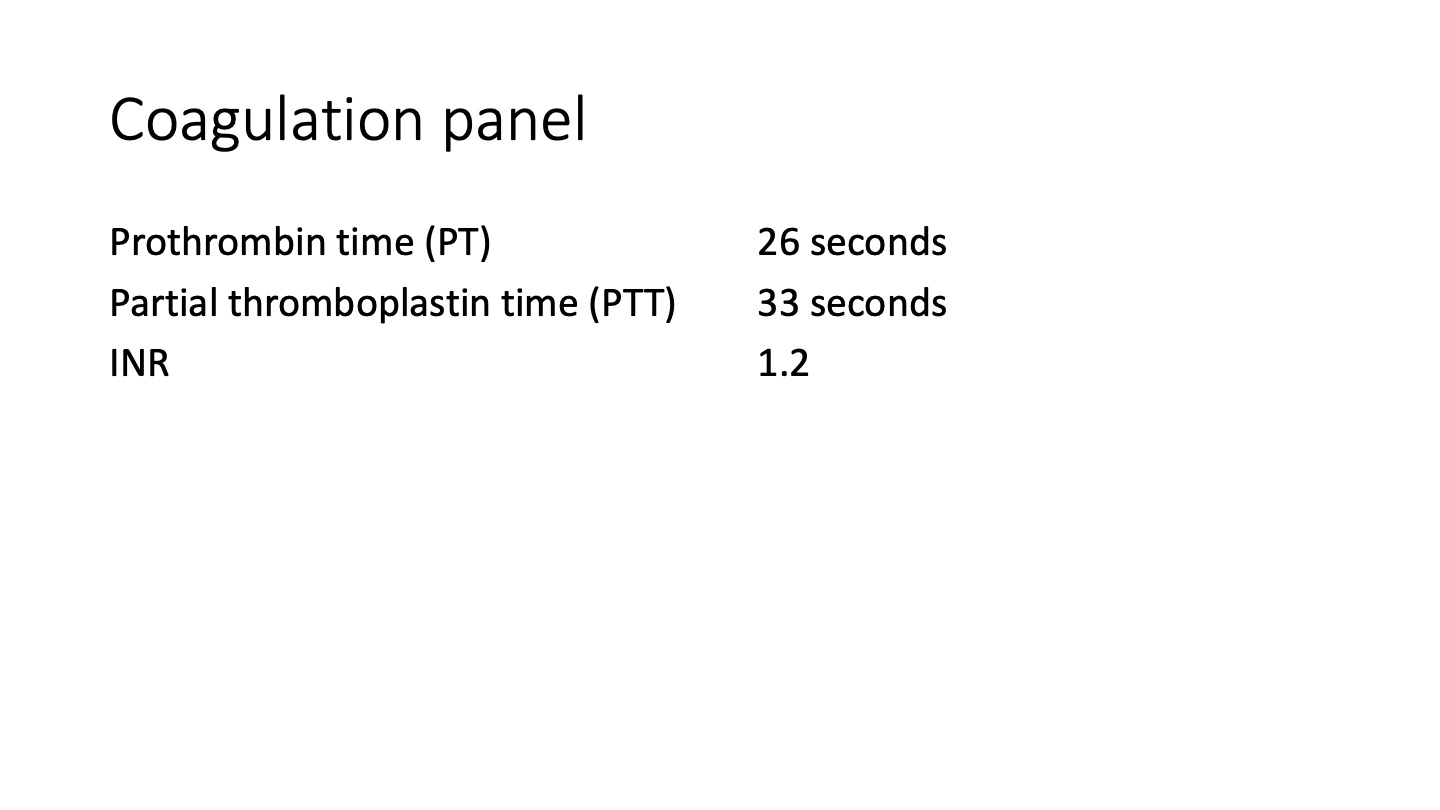

Supplement: Supplementary file 6 [file jetem-8-1-S1-supp6.jpg]

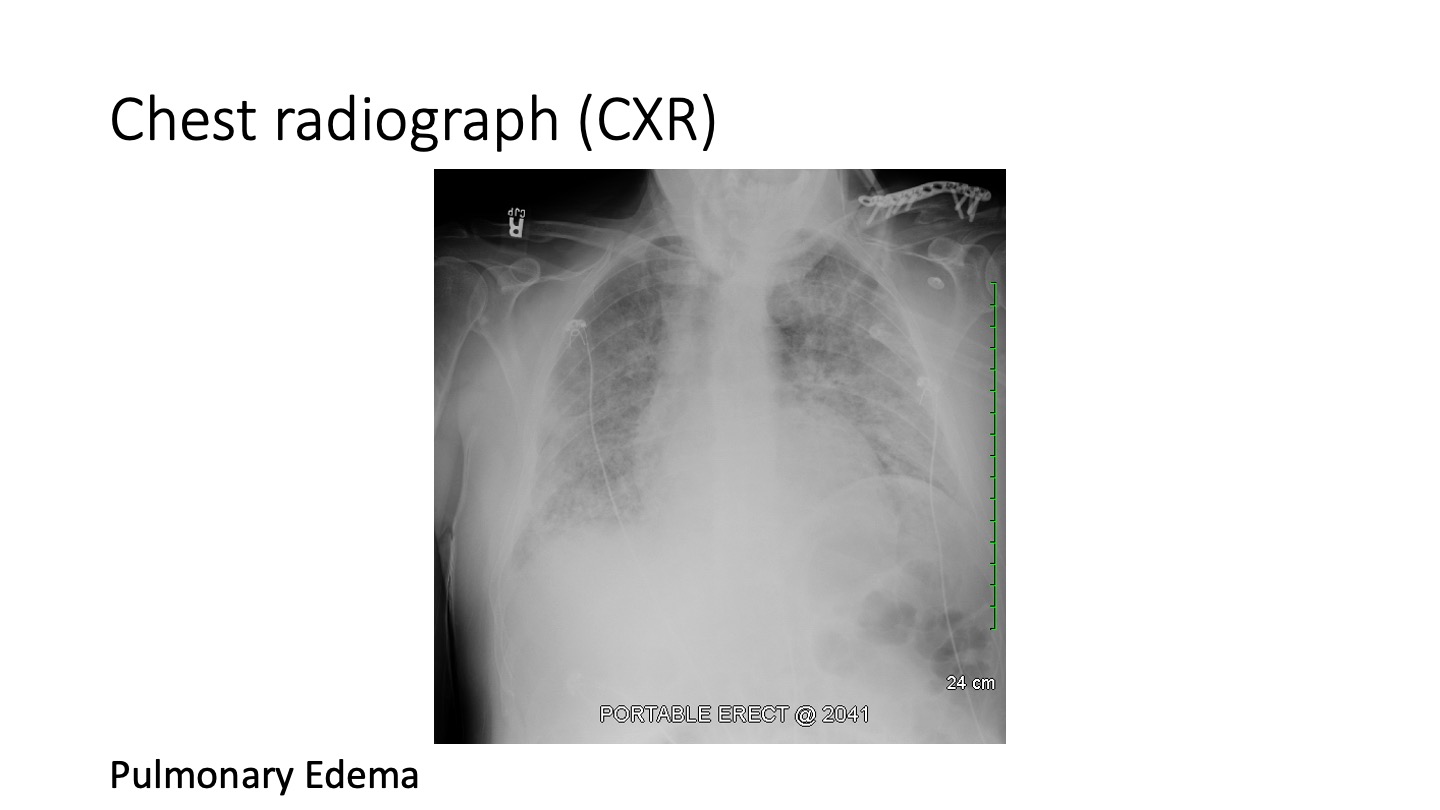

Supplement: Supplementary file 7 [file jetem-8-1-S1-supp7.jpg]

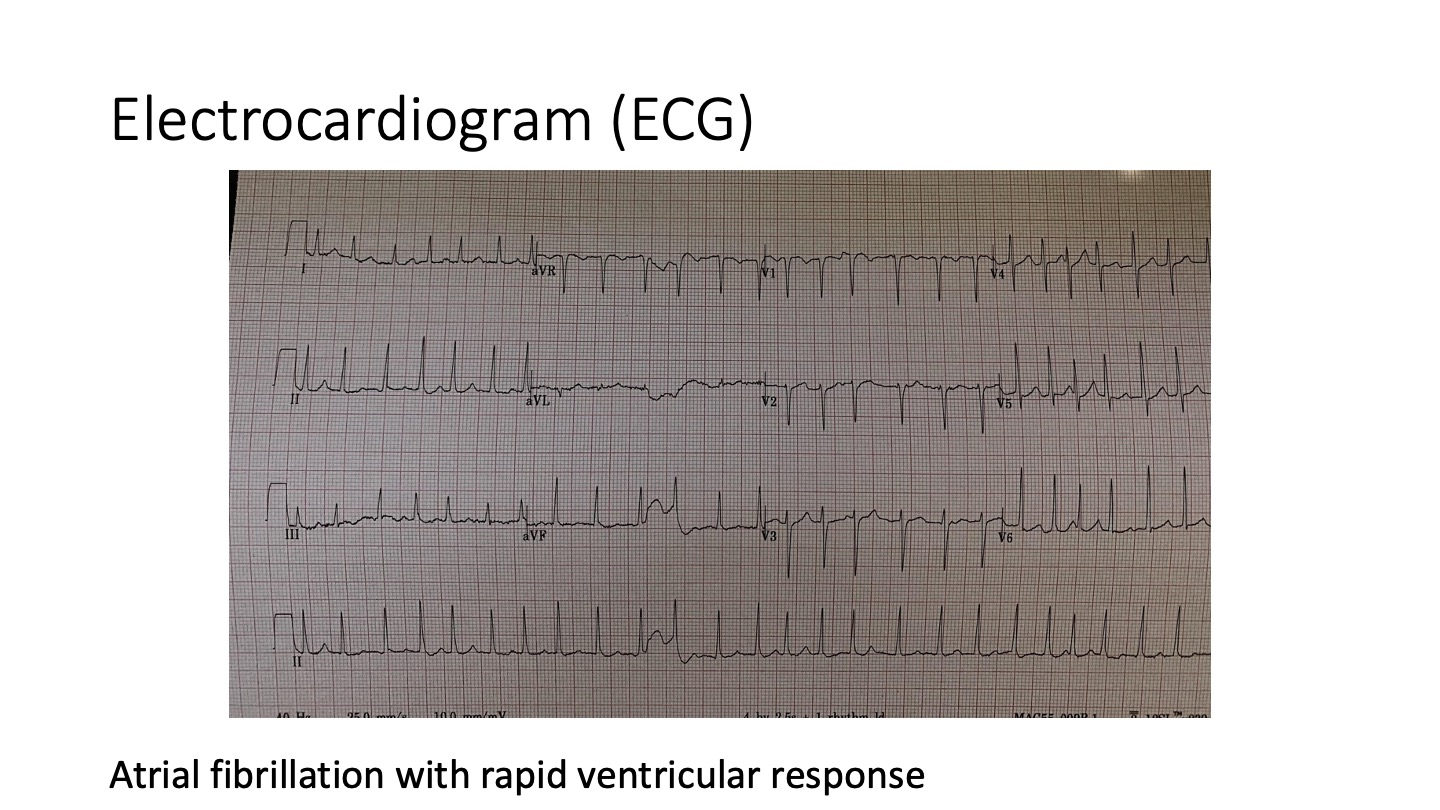

Supplement: Supplementary file 8 [file jetem-8-1-S1-supp8.jpg]

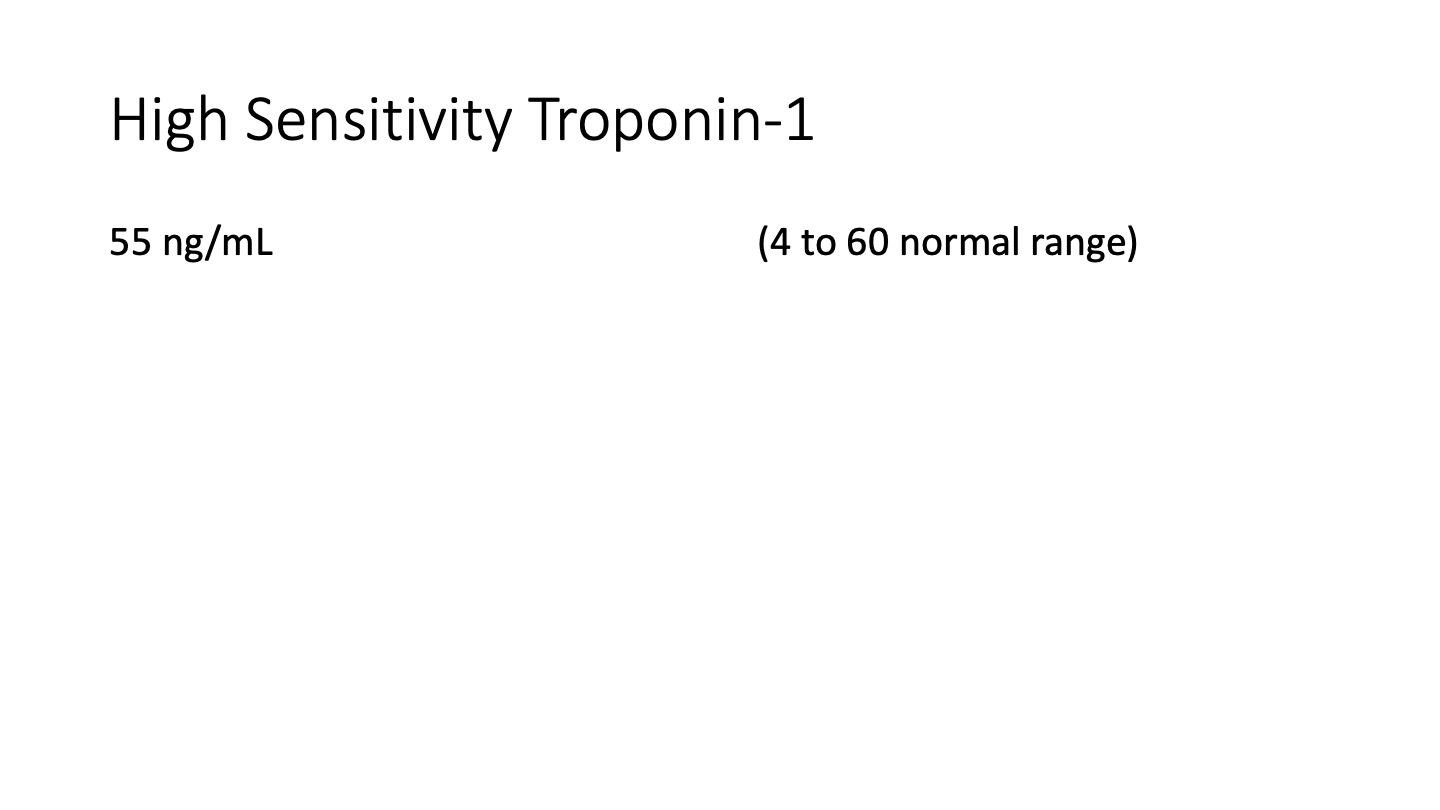

Supplement: Supplementary file 9 [file jetem-8-1-S1-supp9.jpg]
